# Supplementary material for: Long-tailed macaques extract statistical information from repeated types of events to make rational decisions under uncertainty
Source: Sci Rep. 2019 Aug 20;9:12107. doi: 10.1038/s41598-019-48543-0 (PMC6702217; doi:10.1038/s41598-019-48543-0)
Supplement: Supplementary file 1 — Supplementary material [file 41598_2019_48543_MOESM1_ESM.pdf]

Long-tailed macaques extract statistical information from repeated types of events to make rational decisions under uncertainty

Sarah Placì[1,2,3*], Marie Padberg[1,3], Hannes Rakoczy[2,3+], Julia Fischer[1,3+]

[1]Cognitive Ethology Laboratory, German Primate Center, Kellnerweg 4, 37077 Göttingen, Germany

[2]Department of Developmental Psychology, University of Göttingen, Waldweg 26, 37073 Göttingen, Germany

[3]Leibniz ScienceCampus Primate Cognition, German Primate Center, Kellnerweg 4, 37077 Göttingen, Germany

*Corresponding Author: Sarah Placì

[+]Equal contribution

Email: sarah.placi@gmail.com

Phone +49 551 3851-0

Supplementary material

Table S1. Individual participation and performance in each experiment. For each condition of each experiment, we report the number of times, on a total of twelve test trial, that each individual selected the favourable option. Bars indicate conditions in which individuals did not participate.

	**Experiment 1**				**Experiment 2**		**Experiment 3**	
	10/10 vs. 4/10	8/10 vs. 4/10	4/4 vs. 4/10	4/4 vs. 4/10 (side control)	6/6 vs. 6/12	4/4 vs. 4/10	Humans	Boxes
Ilia	10	10	12	11	8	6	5	7
Linus	12	7	7	7	6	5	5	9
Lukas	10	8	4	8	7	6	8	8
Mara	11	10	7	5	9	5	6	7
Mars	11	9	11	8	5	/	6	9
Max	11	12	11	10	9	9	10	7
Maja	10	/	/	6	7	8	6	7
Meiwi	10	8	5	8	8	5	10	5
Mila	11	10	7	9	8	8	9	7
Milka	10	11	8	8	6	8	5	8
Moritz	/	/	/	9	/	6	7	/
Paul	12	/	/	/	/	/	/	/
Sambia	/	/	/	9	10	6	8	6
Snickers	10	11	12	/	9	11	/	/
Sissi	/	/	/	/	/	5	/	/
Smilla	/	/	/	10	/	9	/	/

Experiment 1 - Generalized linear mixed model

We ran a GLMM to assess whether the rewarding frequencies had an effect on monkeys´ performance. As Exp. 1d served as a control to rule out that monkeys only associated the rewarding frequencies with the side of presentation, but that otherwise it had the same rewarding frequencies as Exp. 1c, we did not include it in this analyse. As dependant variable, we used monkeys´ choices (1 for choosing the favourable option, and 0 for choosing the unfavourable one). As fixed factors, we used the condition (Exp. 1a, Exp. 1b and Exp. 1c), the trial number, that we beforehand z-transformed, and the order in which each individual underwent the different condition, which was also z-transformed. As random variable, we used the individual IDs.

The model was the following: Choice ~ Condition + z.Trial + z.Order + (1I Individual)

We used the “vif” function of the “car” package to check for collinearity. There was no collinearity between our predictors, as all values were below 3. Our model was not overdispersed, as the dispersion parameter was 0.856. We used the “drop1” function of the package “lme4” using “Chisq” to calculate the p-value. As we were also interested in testing differences between conditions, we ran a postdoc analyse using the function “glht from the package “multicomp”. Results revealed that condition had a main effect on subjects´ performance, as well as the trial number (see Table S2), and that there was a significant difference between Exp. 1a and Exp. 1c (see Table S3).

Table S2. Results of the generalized linear mixed model.

	Estimate	Std. Error	Chisq	p-value
Intercept	3.374	0.551	*	*
ConditionExp1b	-1.328	0.661	14.457	0.001
ConditionExp1c	-2.311	0.689		
z.Trial	1.640	0.214	94.615	0.000
z.Order	0.137	0.300	0.207	0.649

Table S3. Results of the multicomparison analyse between conditions.

	Estimate	Std. Error	z value	p-value
ConditionExp1b == 0	-1.328	0.661	-2.009	0.10
ConditionExp1c == 0	-2.311	0.689	-3.354	0.002
ConditionExp1b – ConditionExp1c == 0	0.983	0.372	2.64	0.021

Table S4. This table shows the results of the generalised linear mixed models, in which the stay/switch behaviour of the monkeys was the dependant variable, and whether they got a reward or not (Reward) and whether they chose the favourable option (Correct_Choice) were the fixed factors and individuals IDs were included as random factors.

		**Estimate**	**Std. Error**	**z value**	**p-value**
Exp. 1a	Intercept	-2.16	1.15	-1.87	0.06
	Reward	0.11	1.62	0.07	0.94
	Correct_Choice	4.79	1.38	3.46	<.001
Exp. 1b	Intercept	-0.24	0.50	-0.48	0.63
	Reward	0.16	0.61	0.26	0.80
	Correct_Choice	2.54	0.60	4.26	<.001
Exp. 1c	Intercept	0.90	0.57	1.58	0.12
	Reward	-0.22	0.79	-0.27	0.78
	Correct_Choice	2.18	0.80	2.72	<.01
Exp. 1d	Intercept	-0.69	0.37	-1.88	0.06
	Reward	0.11	0.67	0.16	0.88
	Correct_Choice	1.63	0.60	2.70	<.01
Exp. 2a	Intercept	-0.70	0.45	-1.56	0.12
	Reward	0.02	0.64	0.03	0.97
	Correct_Choice	1.02	0.52	1.96	0.05
Exp. 2b	Intercept	-0.80	0.33	-2.40	0.02
	Reward	1.24	0.54	2.30	0.02
	Correct_Choice	-0.15	0.48	-0.31	0.76
Exp. 3a	Intercept	-0.27	0.34	-0.78	0.43
	Reward	-0.50	0.60	-0.83	0.41
	Correct_Choice	1.09	0.55	1.96	0.05
Exp. 3b	Intercept	-0.19	0.36	-0.54	0.59
	Reward	-1.27	0.74	-1.73	0.08
	Correct_Choice	1.74	0.68	2.55	0.01

Alternative strategy

We ran for each condition of each experiment a post-hoc GLMM. We first coded, for each trial, whether individuals stayed with their choice in the next trial, or switched (Strategy variable). This resulted in 11 trials per individuals, as this information was not available for trial 12 (there was no trial 13). We also coded for each trial whether monkeys received a reward or not (Reward variable), and what the favourable choice would have been (Correct choice variable). For each model, we used as dependant variable Strategy, as fixed factors Reward and Correct choice and as random factor the individual IDs.

The model was the following: Strategy ~ Reward + Correct_Choice + (1I Individual).

For each model, we used the “vif” function of the “car” package to check for collinearity. There was no collinearity between our predictors, as all values were below 3. Our models were not overdispersed, as the dispersion parameter were 0.76 for Exp. 1a, 1.04 for Exp. 1b, 0.83 for Exp. 1c, 1.03 for Exp. 1d, 1.00 for Exp. 2a, 1.03 for Exp. 2b, 1.00 for Exp. 3a, and 1.03 for Exp. 3b.
